# Dual Role of Dysfunctional Asc-1 Transporter in Distinct Human Pathologies, Human Startle Disease, and Developmental Delay

**DOI:** 10.1523/ENEURO.0263-23.2023

**Published:** 2023-11-16

**Authors:** Paul Drehmann, Sinem Milanos, Natascha Schaefer, Vikram Babu Kasaragod, Sarah Herterich, Ulrike Holzbach-Eberle, Robert J. Harvey, Carmen Villmann

**Affiliations:** 1 Institute for Clinical Neurobiology, Julius Maximilians University of Würzburg, 97078 Würzburg, Germany; 2 Neurobiology Division, Medical Reserach Council Laboratory of Molecular Biology, Cambridge CB2 0QH, United Kingdom; 3 Center for Pediatrics and Adolescent Medicine, Pediatric Neurology, Social Pediatrics and Epileptology, University Hospital Gießen, 35392 Giessen, Germany; 4 School of Health, University of the Sunshine Coast, Sippy Downs, QLD 4558, Australia; 5 Sunshine Coast Health Institute, Birtinya, QLD 4575, Australia

**Keywords:** Asc-1 transporter, candidate gene, glycine receptor, glycine uptake, human startle disease, NMDAR

## Abstract

Human startle disease is associated with mutations in distinct genes encoding glycine receptors, transporters or interacting proteins at glycinergic synapses in spinal cord and brainstem. However, a significant number of diagnosed patients does not carry a mutation in the common genes *GLRA1*, *GLRB*, and *SLC6A5*. Recently, studies on solute carrier 7 subfamily 10 (*SLC7A10*; Asc-1, alanine-serine-cysteine transporter) knock-out (KO) mice displaying a startle disease-like phenotype hypothesized that this transporter might represent a novel candidate for human startle disease. Here, we screened 51 patients from our patient cohort negative for the common genes and found three exonic (one missense, two synonymous), seven intronic, and single nucleotide changes in the 5′ and 3′ untranslated regions (UTRs) in Asc-1. The identified missense mutation Asc-1^G307R^ from a patient with startle disease and developmental delay was investigated in functional studies. At the molecular level, the mutation Asc-1^G307R^ did not interfere with cell-surface expression, but disrupted glycine uptake. Substitution of glycine at position 307 to other amino acids, e.g., to alanine or tryptophan did not affect trafficking or glycine transport. By contrast, G307K disrupted glycine transport similar to the G307R mutation found in the patient. Structurally, the disrupted function in variants carrying positively charged residues can be explained by local structural rearrangements because of the large positively charged side chain. Thus, our data suggest that *SLC7A10* may represent a rare but novel gene associated with human startle disease and developmental delay.

## Visual Abstract

**Figure:**
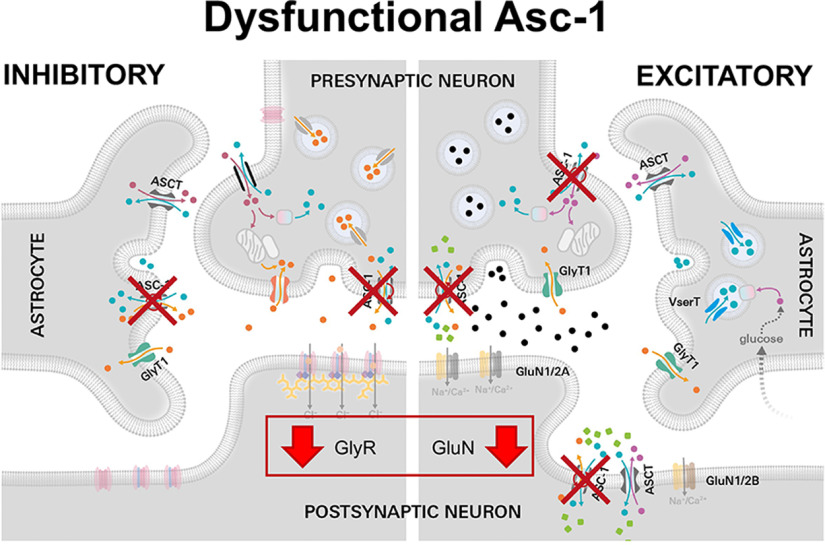

## Significance Statement

Patients diagnosed with startle disease/hyperekplexia do not always carry mutations in the known associated disease genes (*GLRA1*, *GLRB*, *SLC6A5*). Recently, murine studies suggested the solute carrier 7 subfamily 10 (*SLC7A10*) as candidate gene for human startle disease. Within our patient cohort, we found one missense mutation, three exonic silent mutations but also intronic single nucleotide variations (SNVs) as well as variations in the 5′ and 3′ untranslated regions (UTRs) in *SLC7A10*. We further concentrated on the structural and functional alterations because of identified missense mutation in the alanine-serine-cysteine transporter (Asc-1). The exchange of Gly307 with positively charged residues leads to structural rearrangements accompanied by loss of function. Our data provide evidence that in humans *SLC7A10* is a rare gene associated with human startle disease.

## Introduction

Knock-out (KO) mice for solute carrier 7 subfamily 10 (*Slc7a10*), encoding the transporter alanine-serine-cysteine transporter (Asc-1) exhibit a startle disease-like phenotype (Safory et al., 2015). Asc-1 is a sodium-independent plasma membrane transporter present in neurons and astrocytes with high affinity for neutral small amino acids such as glycine, L-serine, D-serine, alanine, and cysteine (Rosenberg et al., 2013; Ehmsen et al., 2016; Sason et al., 2017; Mesuret et al., 2018). *Slc7a10* KO mice display decreased glycine levels in the brain. Moreover, behavioral analysis revealed a phenotype consistent with defective glycinergic transmission, e.g., tremors, hind-leg clasping, increased righting time, and less open field activity (Xie et al., 2005; Safory et al., 2015). The Asc-1 transporter is widely distributed in the rodent brain (Helboe et al., 2003) but in caudal brainstem regions and the spinal cord, Asc-1 is abundant in areas of glycinergic activity. A role of Asc-1 in the maintenance of presynaptic neuronal glycine levels has been suggested (Ehmsen et al., 2016) and consistent with the role of the GlyR α3 subunit in rhythmic breathing, Asc-1 is able to alter respiratory pattern formation (Manzke et al., 2010; Mesuret et al., 2018). Besides its importance for glycinergic neurotransmission, Asc-1 modulates NMDA receptor (NMDAR) activity and thus synaptic plasticity at excitatory synapses (Rosenberg et al., 2013; Sason et al., 2017; Neame et al., 2019).

Asc-1 belongs into the superfamily of heteromeric amino acid transporters (HATs). HATs are important in human health and have been linked to amino acidurias (e.g., cystinuria), tumor cell growth, and glioma invasion (Fotiadis et al., 2013). They are composed of two subunits, a heavy chain (*SLC3* family) and a light chain [L-type amino acid transporter (LAT) *SLC7*; Verrey et al., 2004; Fotiadis et al., 2013]. The heavy chain (4F2hc) is essential for trafficking of the light chain toward the cellular membrane whereas the light chain (Asc-1) harbors the transporter activity (Rosell et al., 2014). The extracellular domain of 4F2hc almost completely interacts with the extracellular face of the light chain Asc-1 and thus stabilizes the heterodimer protein (Rosell et al., 2014). The light chain represents a 12 transmembrane domain (TM) protein linked to the heavy chain by a disulfide bridge (Rosell et al., 2014; Errasti-Murugarren et al., 2019). The crystal structure of Asc-1 is not known yet, but the structure of a homologous bacterial alanine-serine, cysteine exchanger (BasC) was recently solved. This structure provided insights to key determinants important for the asymmetric substrate interaction. TM1-5 stabilize the inward-open conformation, while TM1-5 and TM8 together allow conformational transitions toward the inward-occluded transporter conformation (Errasti-Murugarren et al., 2019; Mikou et al., 2020).

The current understanding of human startle disease (hyperekplexia) is based on mutations in genes encoding glycine receptor subunits (*GLRA1*, *GLRB*), the glycine transporter GlyT2 (*SLC6A5*) as well as in rare cases the synaptic scaffolding proteins collybistin (*ARHGEF9*) and gephyrin (*GPHN*) that lead to significant muscle hypertonia and neonatal apnea episodes (Rees et al., 2002, 2003, 2006; Harvey et al., 2004; Chung et al., 2013; Bode and Lynch, 2014; Schaefer et al., 2022). Our patient cohort contains individuals diagnosed with hyperekplexia that do not apparently harbor mutations in the common genes described.

To assess the possible role of Asc-1 in startle disease, we screened genomic DNA from 51 unresolved hyperekplexia patients for variants in all 11 exons including flanking intronic sequences of the human gene (*SLC7A10*). We discovered three exonic single nucleotide variants, one missense and two synonymous changes. In addition, we detected several intronic nucleotide exchanges as well as variations in 5′ and 3′ UTRs. The missense mutation G307R was further functionally investigated together with other artificial Asc-1 variants at the same position. Our data together with the patient history show that human Asc-1 represents a novel rare candidate gene for human hyperekplexia. However, the accompanying developmental delay in this proband also likely reflects the role of Asc1 at excitatory synapses in the central nervous system.

## Materials and Methods

### Screening procedure

A total of 51 patient samples negative for mutations in the common genes *GLRA1*, *GLRB*, and *SLC6A5* were selected according for a screening approach of *SLC7A10* mutations. As characteristics, we used typical symptoms of human hyperekplexia (e.g., enhanced startle response and muscle rigidity on tactile or acoustic stimuli after birth or in early childhood) and excluded patients which showed other symptoms pointing to a mosaic phenotype or inconsistent with the behavioral phenotype of *Slc7a10* knock-out mice, e.g., epilepsy or heart failure. The study was approved by the Ethics committee of the Julius Maximilians University of Würzburg (2016011301).

### Isolation of genomic DNA

Genomic DNA was isolated from patient blood samples using the “Genomic DNA from blood samples kit” from QIAGEN following the instructions by the supplier. Following determination of the concentration of the genomic DNA, DNA was used for PCR amplification of all 11 exons of *SLC7A10* including 5′ and 3′ intronic regions of the appropriate exons according to use of intronic primer pairs.

### PCR amplification and amplimer purification

PCR conditions used were adapted for each primer pair with varying annealing temperatures between 55–63°C. Generated PCR fragments were verified by gel electrophoresis and purified from agarose gels with the Gel extraction kit (QIAGEN).

### Sequencing and database analysis

The purified PCR amplimers were sequenced (LGC Genomics) in sense and antisense direction and analyzed using the wild-type (WT) Asc-1 sequence as a template. Furthermore, genome databases (gnomAD, https://gnomad.broadinstitute.org; Clinvar, https://www.ncbi.nlm.nih.gov/clinvar/) were used to identify common single nucleotide variations (SNVs) in the human population.

### Molecular cloning

Mutations G307R, G307W, G307K, and G307A were introduced into Asc1 cDNA by overlap extension PCR mutagenesis. The resulting clones were verified by sequencing and used for functional analysis.

### Cell lines

HEK293 cells (human embryonic kidney cells) were purchased from ATCC (CRL-1573; ATCC – Global Biosource Center) and grown in Earle’s minimal essential medium (MEM) supplemented with 10% fetal calf serum, 2 mm GlutaMAX, 1 mM sodium pyruvate and 1 U/ml penicillin, and 100 μg/ml streptomycin (Sigma-Aldrich) under standard growth conditions at 37°C and 5% CO_2_.

### Transfection of cells

For protein expression analysis, HEK293 cells were transiently transfected using a modified calcium-phosphate precipitation method. Here, a mixture of plasmid DNAs encoding for Asc-1 WT (wild-type refers to full-length unmodified Asc-1) or Asc-1-variants (Asc-1^G307R^, Asc-1^G307W^, Asc-1^G307K^, Asc-1^G307A^) together with the heavy chain (4F2hc) were prepared in a 1:1 ratio (1 μg each for 3-cm dishes with 200,000 cells; 10 μg each for 10-cm dishes seeded with 1.8 × 10^6^ HEK293 cells). The DNA mixtures were mixed up with 2.5 m CaCl_2_, 0.1× TE buffer and HBS buffer (50 mm HEPES, 12 mm glucose, 10 mm KCl, 280 mm NaCl, 1.5 mm Na_2_HPO_4_, pH 6.98). The transfection solutions were applied to cell media. After 6 h, the medium was replaced by fresh medium to reduce transfection stress.

For the uptake assay, HEK293 cells were transfected using Lipofectamine 2000 (Invitrogen) according to the manufacturer’s protocol. For this assay, HEK293 cells were grown on gelatin-coated 96-well plates to ensure proper adherence during multiple washing steps required during the uptake assay. All assays were performed 48 h after transfection and eGFP plasmid served as control for transfection efficiency.

### Protein lysates

HEK293 cells grown in 10-cm dishes were transiently transfected with Asc-1 WT or Asc-1-variants together with 4F2hc. Cells were washed twice with PBS followed by incubation with 1 ml *Cytobaster* protein extraction solution (Merck Millipore) supplemented with protease inhibitors (Roche Diagnostics). The cell suspension was centrifuged for 5 min at 16,000 × *g* and 4°C resulting in a supernatant containing solubilized proteins. A total of 20 μg of protein was added to a Laemmli loading buffer containing β-mercaptoethanol. The samples were incubated for 30 min at 37°C before separation by SDS-PAGE (11% polyacrylamide gels).

### Protein biochemical immunodetection

After blotting, the nitrocellulose membrane (GE Healthcare) was blocked for 1 h with 5% BSA in TBS-T (Tris-buffered saline with 1% Tween 20). Primary antibodies such as rabbit-anti Asc-1 diluted 1:500 (ab122751, Abcam), rabbit-anti pan-cadherin diluted 1:1000 (4068S, Cell Signaling), and mouse-anti GAPDH diluted 1:1000 (CB1001, Calbiochem) were incubated overnight at 4°C. Secondary antibodies were HRP-conjugated and anti-mouse or anti-rabbit (Dianova), diluted 1:10,000 in blocking solution. Signals were detected using the ECLplus system (GE Healthcare).

### Biotinylation assay

Biotinylation of surface proteins was performed on transfected HEK293 cells transiently expressing Asc-1 WT or Asc-1^G307R^ together with 4F2hc. 48 h after transfection, medium was removed and cells were washed three times with ice-cold PBS, pH 8.0 (GE Healthcare). The surface proteins were labeled by incubating the cells for 30 min with 1 mg/ml EZ-Link Sulfo-NHS-LC-biotin (sulfonosuccinimidyl-6-(biotin-amido)-hexanoate (Pierce Biotechnologies), followed by incubation with quenching buffer (192 mm glycine and 25 mm Tris in PBS, pH 8.0) for 10 min. Cells were detached using ice-cold PBS buffer followed by centrifugation for 10 min at 1000 × *g*. The resulting pellet was lysed using TBS supplemented with 1% Triton X-100 (Carl-Roth) and a protease inhibitor mixture tablet (Roche Diagnostics). After centrifugation for 1 min at 13,000 × *g*, 100 μl of the supernatant was kept as whole protein fraction. The remaining supernatant was incubated with 50 μl of streptavidin-agarose beads (Pierce Biotechnologies) for 2 h at 4°C while rotating. Then, beads were spun down and the supernatant was removed (intracellular fraction). The remaining beads coupled with biotinylated surface proteins were washed three times using TBS buffer. Biotinylated proteins were detached from beads by boiling the samples in 60 μl of Laemmli buffer for 5 min at 95°C (surface fraction). Twenty micrograms of the whole-cell protein or 40 μl of the surface fraction were analyzed.

### Immunocytochemical stainings

In addition to transfected Asc-1 WT or the mutant Asc-1^G307R^ together with 4F2hc in a 1:1 ratio (each plasmid 1 μg), 0.5 μg of pdsRed-MEM plasmid encoding a fusion protein of red fluorescent protein (dsRed) and a signal sequence of neuromodulin (GAP-43; Clontech) was cotransfected to label the plasma membrane of the transfected cells. Cells were fixed with 4% paraformaldehyde, blocked for 1 h with 5% normal goat serum in PBS and stained with or without permeabilization using 0.1% Triton X-100 for 30 min. Primary antibody incubation (rabbit-anti Asc-1, ab122751, Abcam) lasts for 1 h at room temperature, followed by goat-anti rabbit Alexa488, Dianova) for 30 min. DAPI (1:20,000) was used to stain cell nuclei. Cover slips were mounted with Mowiol (Sigma-Aldrich).

### Confocal microscopy, image acquisition, and analysis

Images were acquired with an inverted IX81 microscope equipped with a FV1000 confocal laser scanning system, a FVD10 SPD spectral detector and diode lasers of 405 nm (DAPI), 495 nm (Alexa488), and 550 nm (Cy3; Olympus). All images shown were acquired with an Olympus UPLSAPO 60× (oil, numerical aperture: 1.35) objective. The images were further developed and organized by Adobe Photoshop and Illustrator software (Adobe).

### Uptake assays

Transfected HEK293 cells grown in gelatin-coated 96-well plates were carefully washed three times with PBS. The uptake assay was performed 48 h post-transfection as follows: [^3^H] glycine was used in a concentration series of 0, 25, 50, 100, 250, 500, 750, 1000 nm in sodium free HBSS medium. Each well was provided with 80 μL of the appropriate dilution (duplicates were always used) and incubated for 10 min at room temperature. Cells were again washed three times with PBS and lysed with ice-cold water (150 μl). A total of 120 μl of cell lysate was used for determination of the glycine uptake; 20 μl were used for protein concentration analysis required to back calculate the uptake rate. Vials containing lysed cells and scintillation solution (Carl-Roth) were counted for 2 min each using a scintillation counter (TriCarb, PerkinElmer). For competing experiments with D-isoleucine (D-ile), a transportable inhibitor for glycine uptake, 4 mm of D-ile was used and co-incubated with various [^3^H] glycine concentrations.

### Statistical analysis

GraphPad Prism 8.3.0 (GraphPad Software) was used to calculate mean values, SD, SEM, and values for statistical significance. Statistical significance was estimated using unpaired *t* tests or one-way ANOVA tests for multiple comparisons. Significance was obtained with *p*-values *p < 0.05, **p < 0.01, ***p < 0.001, ****p < 0.0001.

### Modelling of mutations

Model predicted by the AlphaFold2 (Jumper et al., 2021) for human *SLC7A10* (Uniprot ID: Q9NS82) was used for the possible structural interpretation of mutations. All *in silico* mutations were conducted in Coot (Emsley and Cowtan, 2004) by choosing the most probable rotamer as well as taking into consideration of clashes with surrounding residues. Figures representing structures were prepared by using UCSF ChimeraX (Pettersen et al., 2021).

## Results

### Identification of *SLC7A10* variations by screening of blood samples from patients diagnosed with startle disease

**Figure 1.**
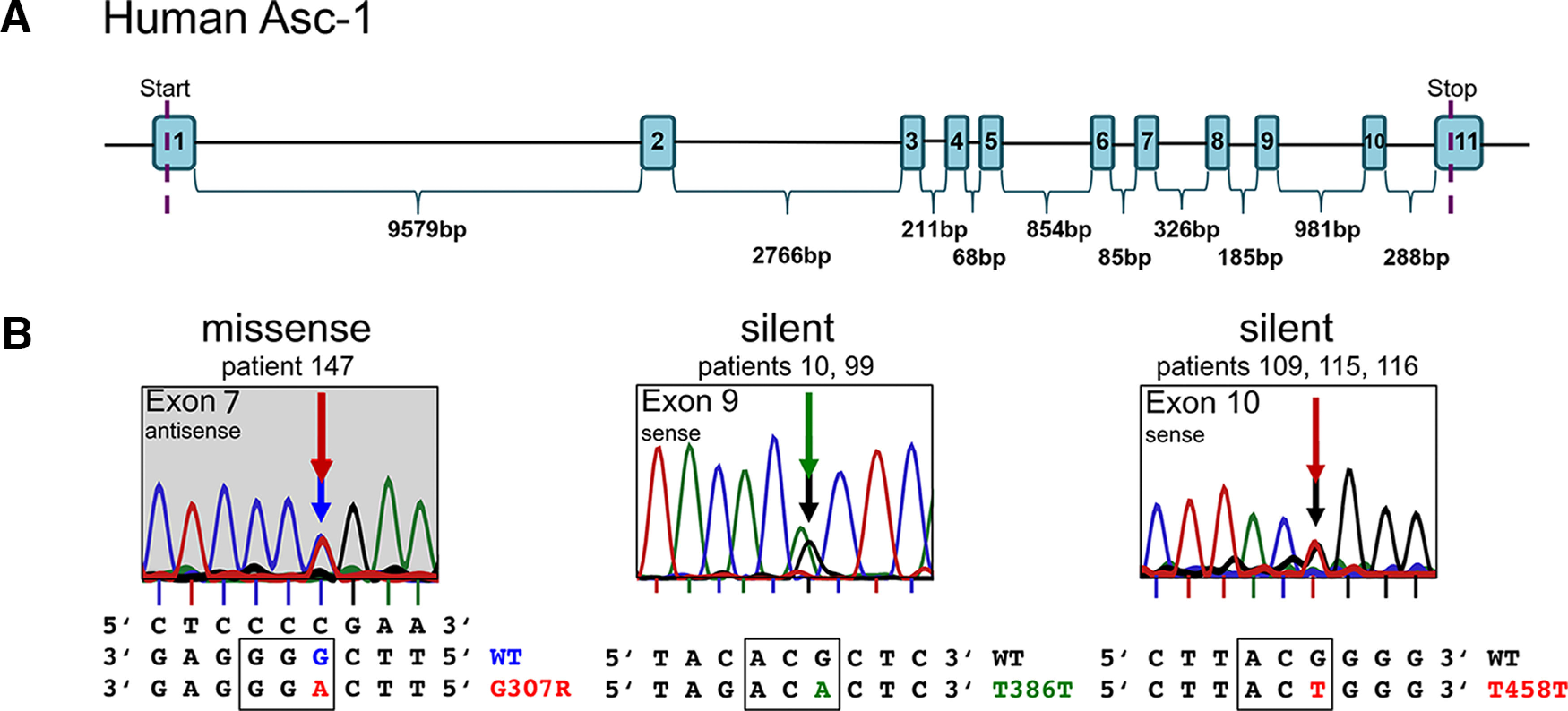
Genomic organization of the human Asc-1 transporter and patient mutations. The *SLC7A10* gene encodes the Asc-1 protein. ***A***, The gene includes 11 exons with long intronic sequences between exons 1–2 and 2–3. ***B***, Screening of 51 patient samples revealed three nucleotide exchanges including one missense mutation in exon 7 c.919G>A; G307R identified in patient 147 and two synonymous mutations c.1158G>A; T386T in exon 9 (patients 10 and 99) and c.1374G>T; T458T in exon 10 (patients 109, 115, 116).

We screened 51 patients from our startle disease cohort with so far unknown origin of the disease and negative for *GLRA1*, *GLRB*, and *SLC6A5* for all 11 exons and adjacent intronic regions of *SLC7A10*, encoding Asc-1. Patients matched the following criteria: typical phenotype of hyperekplexia with enhanced startle response and muscle rigidity on tactile or acoustic stimuli certainly after birth or in early childhood. Sequencing of the GlyR subunit genes as well as the GlyT2 transporter were negative. We discovered three exonic single nucleotide variants generating one missense mutation in exon 7 c.919G>A; G307R and two synonymous changes c.1158G>A; T386T in exon 9 and c.1374G>T; T458T in exon 10 (Fig. 1). All of them represented as heterozygous with one functional copy of Asc-1 remaining.

In addition, we detected seven intronic nucleotide variations that could have an impact on splicing processes or binding of enhancer/silencer elements. Additionally, 5′ UTR and 3′ UTR variations were identified that could influence gene expression by changes in the translation efficiency, stability, and transport of the Asc-1 mRNA (Table 1).

**Table 1:** Screening of patients diagnosed for hyperekplexia

Localization	Nomenclature	Type of mutation	Missense/silent	Frequency	Disease	SNV number	Identification
**5′ UTR, Exon 1**	**c.−23C>A**	**SNV**		**1/51**	**Hyperekplexia**		**Own study**
**Intron 2**	**c.356 + 138G>C**	**SNV**		**6/51**	**Hyperekplexia**		**Own study**
**Intron 2**	**c.356 + 162C>G**	**SNV**		**1/51**	**Hyperekplexia**		**Own study**
**Intron 3**	**c.508 + 51T>C**	**SNV, common**		**37/51**	**Hyperekplexia**		**Own study**
**Intron 4**	**c.634 + 48C>G**	**SNV**		**8/51**	**Hyperekplexia**		**Own study**
**Intron 5**	**c.788 + 95G>A**	**SNV, common**		**39/51**	**Hyperekplexia**		**Own study**
**Intron 6**	**c.913-25T>C**	**SNV, splice site**		**10/51 (0.1960)**	**Hyperekplexia**		**Own study**
**Intron 6**	**c.913-11T>C**	**SNV, splice site**		**35/51 (0.68)**	**Hyperekplexia**		**Own study**
	c.913-4C>A	SNV, splice site		0.0000008	No association	rs747357569	gnomAD
	c.913-5C>T	SNV, splice site		0.00003186	No association	rs372803928	gnomAD
	c.913-7C>T	SNV, splice site		0.0000008	No association	rs756877434	gnomAD
	c.913-8C>T	SNV, splice site		0.0000008	No association	rs527258250	gnomAD
	c.913-2_915delAGACC	SNV, splice acceptor site		0.000003979	No association	rs1296796950	gnomAD
**Exon 7**	**c.919G>A**	**Missense**	**G307R**	**1/51**	**Hyperekplexia**		**Own study**
	c.920G>A	Missense	G307E	single case	No association	rs1395052005	gnomAD, ClinVar
**Exon 9**	**c.1158G>A**	**Synonymous**	**T386T**	**2/51 (0.039)**	**Hyperekplexia**		**Own study**
	c.1158G>A	Synonymous	T386T	0.0126–0.01149	No association	rs2303094	gnomAD
	c.1157C>T	Missense	T386M	0.0000008	No association	rs762156060	gnomAD
	c.1156A>C	Missense	T386P	0.000004025	No association	rs772026103	gnomAD
	c.1156A>G	Missense	T386A	0.0000008	No association	rs772026104	gnomAD
**Exon 10**	**c.1374G>T**	**Synonymous**	**T458T**	**3/51 (0.058)**	**Hyperekplexia**		**Own study**
	c.1374G>T	Synonymous	T458T	0.045–0.055	No association	rs2303094	gnomAD
	c.1374G>C	Synonymous	T458T	0.00003580	No association	rs2303094	gnomAD
	c.1374G>A	Synonymous	T458T	0.00002829	No association	rs2303094	gnomAD
	c.1374G>GT	Frameshift	Thr458AsnfsTer71	0.00001193	No association	rs750357075	gnomAD
**3′ UTR, exon 11**	c.*131_132insT	**Common insertion, frameshift**		**45/51**	**Hyperekplexia**		**Own study**
**3′ UTR, exon 11**	c.*188C>T	**SNV**		**3/51**	**Hyperekplexia**		**Own study**
**3′ UTR, exon 11**	c.*262T>C	**SNV, common**		**39/51**	**Hyperekplexia**		**Own study**

SNV, single nucleotide variation; UTR, untranslated region. Bold content refers to missense mutation of own study.

The novel identification of the missense mutation Asc-1^G307R^ has not been reported to date (and is absent in gnomAD and ClinVar databases) and has a Combined Annotation Dependent Depletion (CADD) score of 32 (damaging; Rentzsch et al., 2019). The gnomAD database documented another amino acid exchange at position 307, c.920G>A, p.G307E for a single case in a total of 31 382 genomes with an allele frequency of 0.00003187 (rs1395052005) and a CADD score of 28. However, no disease association was reported for p.G307E to date in ClinVar or elsewhere. The intronic and UTR SNPs which underwent a careful *in silico* analysis are present in gnomAD and ClinVar, however they are therefore unlikely to be pathogenic. Variant Asc-1^T458T^ (three out of 51 patients) seems to be a common SNV in the human population with allele frequencies of 0.05–0.55 (15552/282744, gnomAD, rs2303094). Other synonymous mutations at position T458 have been reported, however less frequent (Table 1). In our cohort, the Asc-1^T386T^ variant was detected in two out of 51 patients. Asc-1^T386T^ is common in humans with 3215/279690 with 41 homozygous cases (rs150600610) in gnomAD. Other missense mutations have been reported at T386 with substitutions into alanine (6/279828; frequency 0.00002144; rs772026104), proline (1/248454, frequency 0.000004025, rs772026103), and methionine (4/249392, frequency 0.00001610, rs762156060) in heterozygous cases only (Table 1, gnomAD). Again, no disease association has been reported.

#### Clinical description of the patient (SLC7A10 G307R)

This male child was referred from a district hospital by the local pediatrician for assessment of startle disease/hyperekplexia. He was born at 40 weeks of gestation by spontaneous vaginal delivery. During the first month of life, an unclear muscular hypertonia was observed. He showed massive tremor in arms and legs also during nights. The observed stiffness was less prominent following the third month of life, however exaggerated startle responses and hypertonia persisted on unexpected noise or touch. At the age of two years, developmental delay became obvious concomitant with a delay in language development. At the age of 10 years, he exhibited still a developmental delay with an IQ of 78.

### Trafficking of the Asc1 mutant together with the heavy chain is not altered

The missense mutation G307R of a small glycine into a charged arginine with a long side chain was predicted to be damaging by Combined Annotation Dependent Depletion (CADD) and could affect transporter biogenesis and function. Therefore, Asc-1^G307R^ was coexpressed in HEK293 cells together with the essential heavy chain protein 4F2hc to enable trafficking of the transporter complex toward the cellular surface (Rosell et al., 2014).

**Figure 2.**
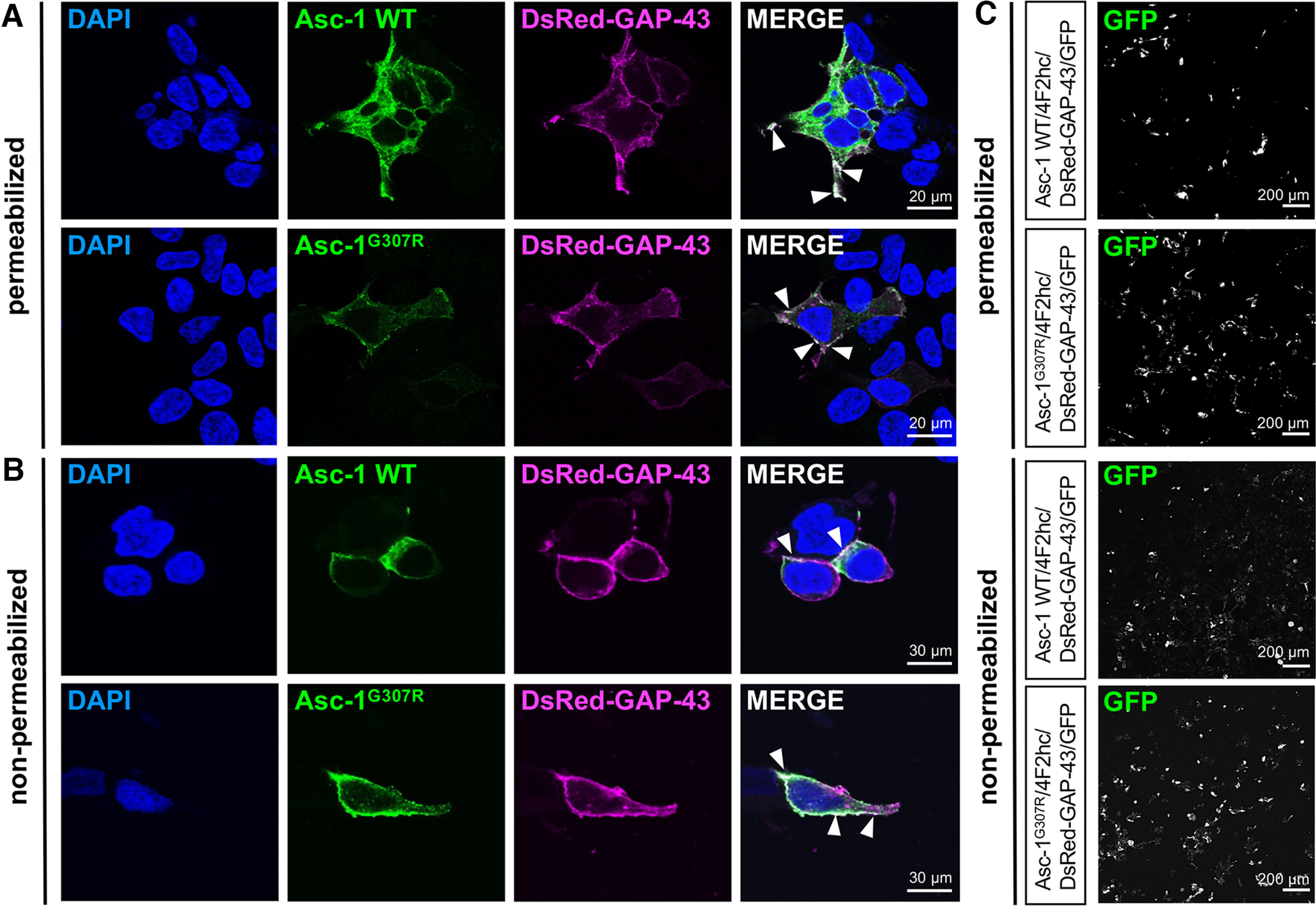
Reduced whole-cell expression of Asc-1^G307R^ does not result in inability to traffic. Asc-1 WT and the variant Asc-1^G307R^ were co-expressed in HEK293 cells with the heavy chain 4F2hc and fusion protein of a neuromodulin sequence (GAP-43) and the fluorescent protein DsRed (DsRed-GAP-43, magenta). ***A***, Forty-eight hours post-transfection, cells were either permeabilized or (***B***) used nonpermeabilized to show membrane expression of Asc-1 WT and the Asc-1 variant Asc-1^G307R^ (green) colocalized with DsRed-GAP-43. Scale bar: 30 μm. White arrow heads point to colocalization of Asc-1 with the membrane marker DsRed-GAP-43. ***C***, Images from a parallel immunostaining at a lower magnification (scale bar: 200 μm) to demonstrate similar transfection efficiencies of the Asc-1 WT and mutant protein. Co-transfected GFP (shown in white) was used as an internal transfection efficiency control.

**Figure 3.**
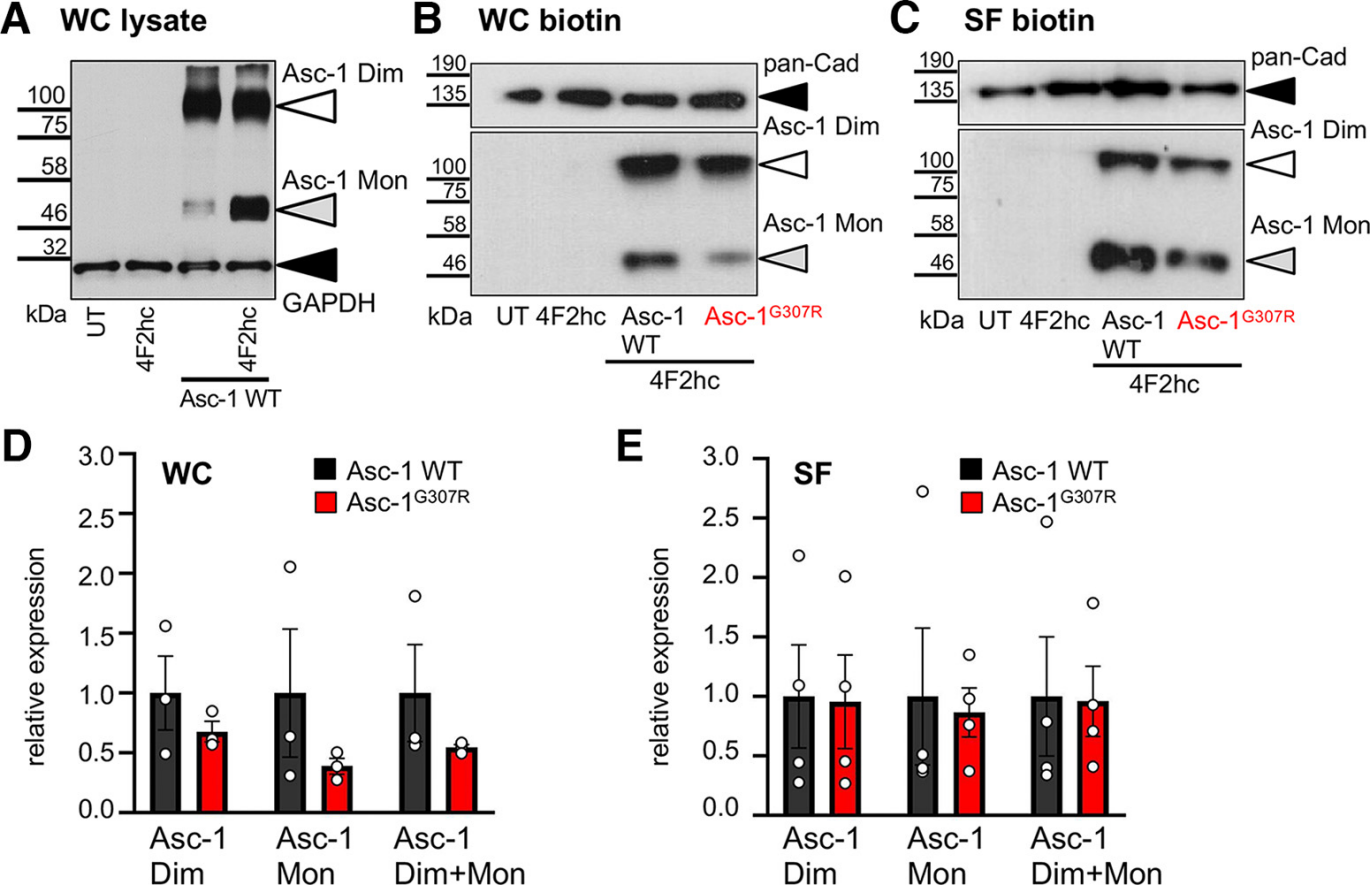
Asc-1^G307R^ is transported highly efficiently toward the cellular surface. ***A***, Asc-1 is specifically stained as monomeric (50 kDa) and dimeric (110 kDa) protein isoform. GAPDH served as housekeeping protein. Asc-1 WT and Asc-1^G307R^ protein level were quantified following biotinylation of surface expressed proteins. ***B***, ***C***, Representative Western blots from whole-cell protein (***B***) and surface-expressed proteins (***C***) immunostained with either the pan-Cad antibody (pan-cadherin; membrane marker) or the Asc-1 antibody. ***D***, Quantification of the Asc-1 whole-cell protein (WC). The expression of Asc-1 was correlated to the marker protein pan-Cad. Asc-1 Mon refers to the Asc-1 monomer (50 kDa), Asc-1 Dim to 110 kDa and both protein bands together (Asc-1 Mon+Dim). ***E***, Quantification of the surface-expressed Asc-1 normalized to the pan-Cad expression. The Asc-1 WT (black) is always compared with the mutant Asc-1^G307R^ (red).

Using permeabilized transfected cells, the mutant Asc-1^G307R^ was expressed at lower levels in the intracellular compartments but prominent at the plasma membrane. The residual and continuous expression of Asc-1 WT in the endoplasmic reticulum because of high overexpression was almost absent from cells transfected with Asc-1^G307R^. Staining of Asc-1 in cells cotransfected with a marker protein (GAP-43) but without permeabilization, exhibited no obvious differences between the Asc-1 WT and Asc-1^G307R^ (Fig. 2*A*,*B*). These observations were not dependent on different transfection efficiencies as seen on cotransfection with GFP (Fig. 2*C*). To quantify transporter expression, transfected HEK293 cells were subjected to biotinylation assays which allow to discriminate between whole-cell and cell-surface protein. Whole-cell protein lysates from transfected cells demonstrated two protein bands detected by the polyclonal Asc-1 antibody. The lower 50-kDa protein represents the Asc-1 monomer. The upper ∼110-kDa protein band represents most likely an Asc-1 dimer (Fig. 3*A*). A high affinity interaction of Asc-1 but also tight association of the 4F2hc heavy chain together with Asc-1 was first proposed by biochemical analysis using different reducing conditions, but also structural analyses provided evidence for this interaction via disulfide bridges (Helboe et al., 2003; Rosell et al., 2014). When compared with the loading control GAPDH, the monomeric Asc-1 protein seems to be reduced in lysates on overexpression of the Asc-1 mutant (Fig. 3*B*). We quantified whole-cell and surface Asc-1 protein cumulative from both protein bands as well as separately to sustain differences in the monomeric Asc-1 (lower molecular weight band Asc-1 Mon) and the high molecular weight Asc-1 protein band (Asc-1 Dim). Cumulative Asc-1^G307R^ expression was not significantly changed with 54 ± 3% whole-cell expression (*p* = 0.3839) and 96 ± 29% plasma membrane (*p* = 0.6854) incorporation compared with WT (Fig. 3*B*,*D*; Table 2). The separate assessment of the monomeric and dimeric Asc-1 protein demonstrated a decrease in the whole-cell expression levels (50 kDa 39 ± 9%, *p* = 0.4241; 110 kDa 68 ± 9%, *p* = 0.3601) similar to the observations from immunocytochemical stainings (Fig. 2). Interestingly, this reduction was not further transmitted to the surface fraction of Asc-1. Although the cumulative plasma membrane expression seemed to be reduced it was not significant neither for the monomeric Asc-1^G307R^ (86 ± 20%, *p* = 0.5284), nor for the larger molecular weight Asc-1 protein band (95 ± 39%, *p* = 0.9230; Fig. 3*C*,*E*). These data argue for an efficient transport to the cellular membrane. Hence, the structural exchange of the small glycine into the arginine carrying a positively charged side chain does not interfere with 4F2hc assembly and exit mechanisms from the endoplasmic reticulum.

**Table 2:** Expression analysis of the human Asc-1 mutant

Asc1 + 4F2hc		Relative expression		Significance	
	MW	Whole cell	Surface	Whole cell #*p*-value	Surface #*p*-value
Asc1 WT	50 kDa	1	1		
	110 kDa	1	1		
	50 + 110 kDa	1	1		
Asc1^G307R^	50 kDa	39 ± 9	86 ± 20	0.4241	0.5284
	110 kDa	68 ± 9	95 ± 39	0.3601	0.9230
	50 + 110 kDa	54 ± 3	96 ± 29	0.3839	0.6854
*n*		3	4	3	4

MW, molecular weight; *n* = number of experiments; #*p*-values were determined from normalized absolute expression.

### Patient Asc-1 variant G307R abolishes function of the Asc-1 transporter

**Figure 4.**
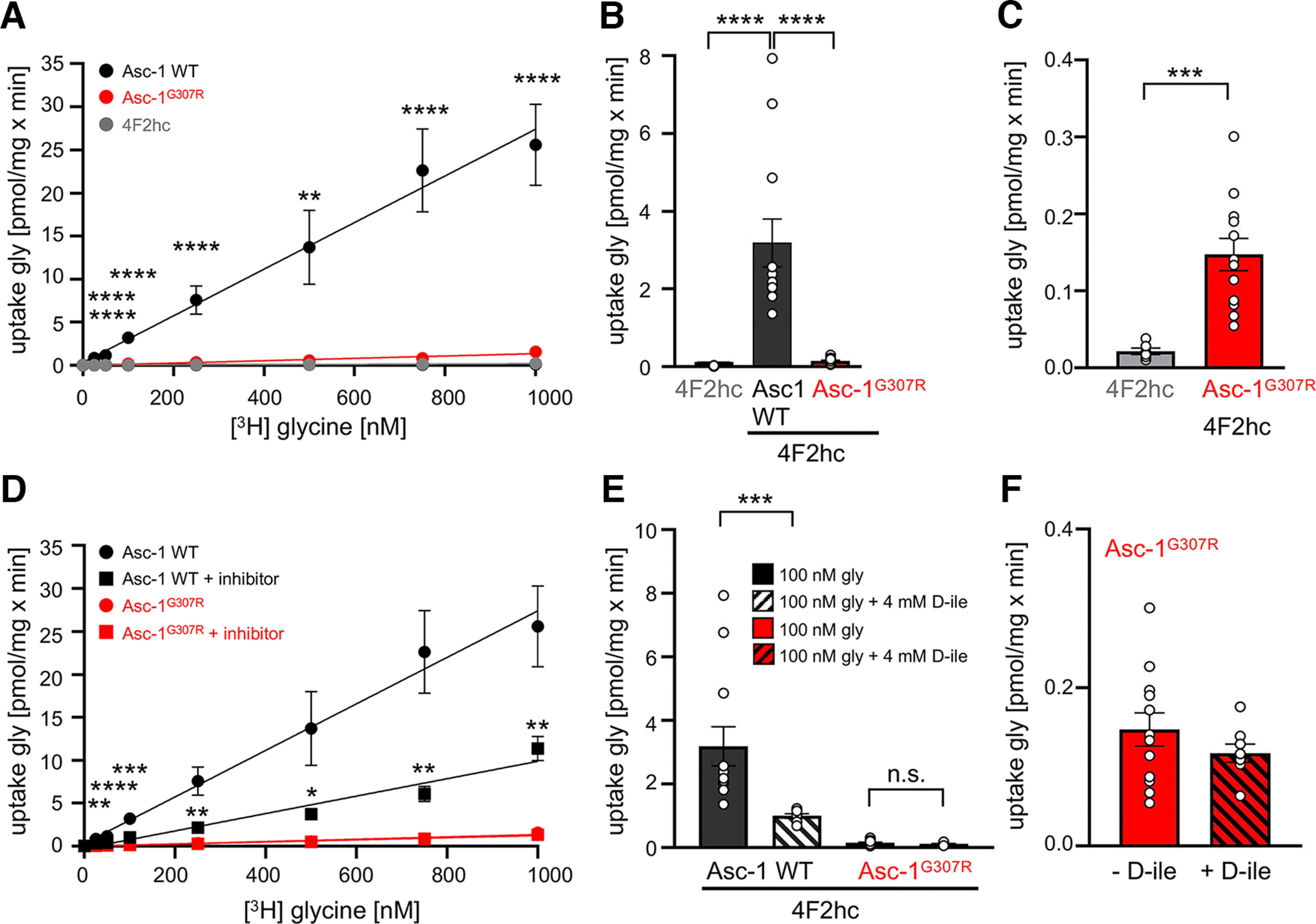
The startle disease mutation Asc-1^G307R^ is highly impaired in transport activity. Glycine uptake assays were used to estimate the transport capacity of the Asc-1^G307R^ variant. ***A***, [^3^H] glycine was used in a concentration series of 0, 25, 50, 100, 250, 500, 750, 1000 nm. Glycine uptake was measured after 10 min. The single expressed 4F2hc heavy chain was used a negative control (gray circle) and compared with Asc-1 WT (black circles) and Asc-1^G307R^ (red circles). ***B***, Glycine uptake at 100 nm glycine. ***C***, Magnification of the glycine uptake values (pmol/mg × min) of the mutant Asc-1^G307R^ (red) and the heavy chain 4F2hc (gray) demonstrating that the mutant although highly impaired in glycine uptake is still significantly different from background level of glycine transport in HEK293 cells. ***D***, Glycine uptake in the presence of the transportable inhibitor D-isoleucine (D-ile). Asc-1 WT transport capability is reduced (gly = black circles; gly+D-ile = black squares) in the presence of the inhibitor while D-ile seems not to affect the mutant variant Asc-1^G307R^ (gly = red circles; gly+D-ile = red squares). ***E***, Comparison of glycine uptake in the presence and absence of the inhibitor D-ile at 100 nm glycine. ***F***, Magnification of the glycine transport in the presence of D-ile at 100 nm glycine. Statistical significance was estimated using the ANOVA test for multiple comparisons with *p < 0.05, **p < 0.01, ***p < 0.001, ****p < 0.0001. All experiments were performed seven times (*n* = 7).

The Asc-1^G307R^ variants did not accumulate in the endoplasmic reticulum and were transported to and integrated into the surface membrane of cells. To investigate the function of the Asc-1 transporter, glycine uptake assays were performed. HEK293 cells were transfected either with 4F2hc alone (uptake threshold) or in combination with Asc-1 WT or Asc-1^G307R^. To circumvent the action of endogenous Asc-1, sodium-free buffers were used up until the assay was performed. The [^3^H] glycine uptake was monitored for 10 min at glycine concentrations between 25 nm and 1000 nm, which showed an almost linear transport up to 1000 nm [^3^H] glycine used (Fig. 4). In contrast to Asc-1 WT, the glycine transport activity of the Asc-1^G307R^ was almost abolished (100 nm gly Asc-1 WT 3.188 ± 0.617 pmol/mg × min, Asc-1^G307R^ 0.147 ± 0.021 pmol/mg × min, *p* < 0.0001, *t* test; Fig. 4*A*,*B*; Table 3). Although the glycine uptake of Asc-1^G307R^ looked similar to the nontransport capability of the 4F2hc heavy chain, the mutant Asc-1^G307R^ revealed a significantly higher transport activity compared with 4F2hc, hence arguing for a significantly impaired transporter function for Asc-1^G307R^ (***p = 0.008, *t* test; Fig. 4*C*). D-isoleucine is a highly efficient competitive inhibitor of glycine at Asc-1 (Rosenberg et al., 2013). Here, we used a 4 mm concentration of D-isoleucine to block the function of glycine uptake by Asc-1 WT or the mutant Asc-1^G307R^. The uptake function of the human Asc-1 WT was blocked by ∼50% (100 nm gly Asc-1 WT 0.993 ± 0.069 pmol/mg × min, Asc-1^G307R^ 0.117 ± 0.011 pmol/mg × min, *p* < 0.0001, one-way ANOVA) whereas the function of the mutant was not affected by D-isoleucine (Fig. 4*D–F*). This might be explained by the very low uptake function of the mutant Asc-1^G307R^ before inhibition by D-isoleucine or the residual uptake is because of low-level of endogenous glycine uptake. A lack of the D-isoleucine block has been observed previously in the Asc-1 KO mouse model (Safory et al., 2015).

**Table 3:** Glycine uptake by the human Asc-1 mutant

	[^3^H] glycine (nm)	Uptake					
		[^3^H] glycine (pmol/mg × min)		Significance	D-isoleucine (pmol/mg × min)		Significance
		Mean ± SEM	*n*	*p*-value compared with Asc1 WT	Mean ± SEM	*n*	*p*-value compared with (-) inhibitor
4F2hc	0	0.000 ± 0.000	6				
	25	0.012 ± 0.005	6	0.0001			
	50	0.012 ± 0.001	6	<0.0001			
	100	0.021 ± 0.004	6	<0.0001			
	250	0.045 ± 0.006	6	0.0001			
	500	0.069 ± 0.007	6	0.0054			
	750	0.130 ± 0.027	6	0.0001			
	1000	0.206 ± 0.068	6	<0.0001			
Asc1 WT	0	0.000 ± 0.000	12		0.000 ± 0.000	8	
	25	0.833 ± 0.175	12		0.336 ± 0.016	8	0.0072
	50	1.148 ± 0.154	11		0.405 ± 0.066	8	<0.0001
	100	3.188 ± 0.617	12		0.993 ± 0.069	8	0.0008
	250	7.583 ± 1.642	12		2.150 ± 0.339	8	0.0018
	500	13.708 ± 4.301	11		3.732 ± 0.625	8	0.0226
	750	22.612 ± 4.816	12		6.072 ± 0.893	8	0.0012
	1000	25.582 ± 4.718	10		11.359 ± 1.387	8	0.0021
Asc1^G307R^	0	0.000 ± 0.000	12		0.000 ± 0.000	8	
	25	0.047 ± 0.005	12	<0.0001	0.042 ± 0.004	8	0.9994
	50	0.068 ± 0.008	12	<0.0001	0.060 ± 0.005	8	0.0074
	100	0.147 ± 0.021	12	<0.0001	0.117 ± 0.011	8	0.9982
	250	0.328 ± 0.038	12	<0.0001	0.268 ± 0.026	8	0.9990
	500	0.526 ± 0.062	12	0.001	0.485 ± 0.046	8	>0.9999
	750	0.814 ± 0.124	12	<0.0001	0.847 ± 0.118	8	>0.9999
	1000	1.563 ± 0. 403	11	<0.0001	1.330 ± 0.282	8	0.0077

*n* = number of independent experiments.

**Figure 5.**
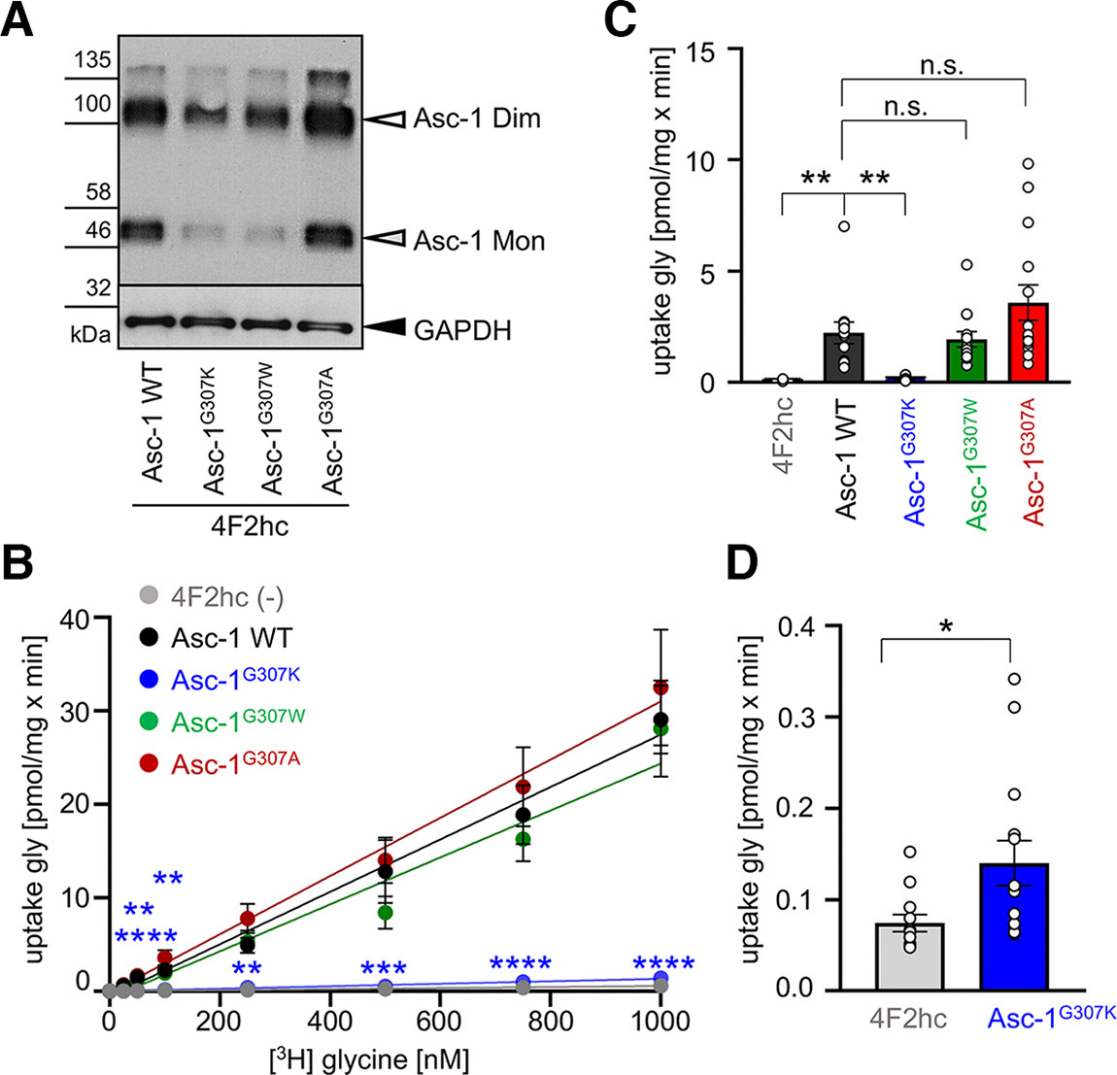
The positively charged side chain of the amino acid at position 307 is responsible for impaired Asc-1 transport activity. ***A***, Protein lysates from cultures co-transfected with Asc-1 variants G307K, G307W (SNV present in the human population, single case described), G307A or Asc-1^WT^ together with 4F2hc. GAPDH was used as positive loading control protein (36 kDa). GFP transfected cells were used as MOCK control. Cells transfected with 4F2hc were used as negative control. ***B***, Glycine uptake assays for artificial Asc-1 variants G307W, G307R, and G307A. Note that the Asc-1^G307K^ is unable to transport glycine while Asc-1^G307W^ and Asc-1^G307A^ retain WT capabilities of glycine transport. ***C***, Glycine uptake a 100 nm glycine demonstrating the significant reduction in glycine transport for mutant Asc-1^G307K^. ***D***, Comparison of the endogenous glycine uptake by the cells without expression of the Asc-1 transporter compared with Asc-1^G307K^. Significance values *p < 0.05, **p < 0.01, ***p < 0.001, ****p < 0.0001. Five experiments have been performed (*n* = 5), all probes were run in duplicates.

**Figure 6.**
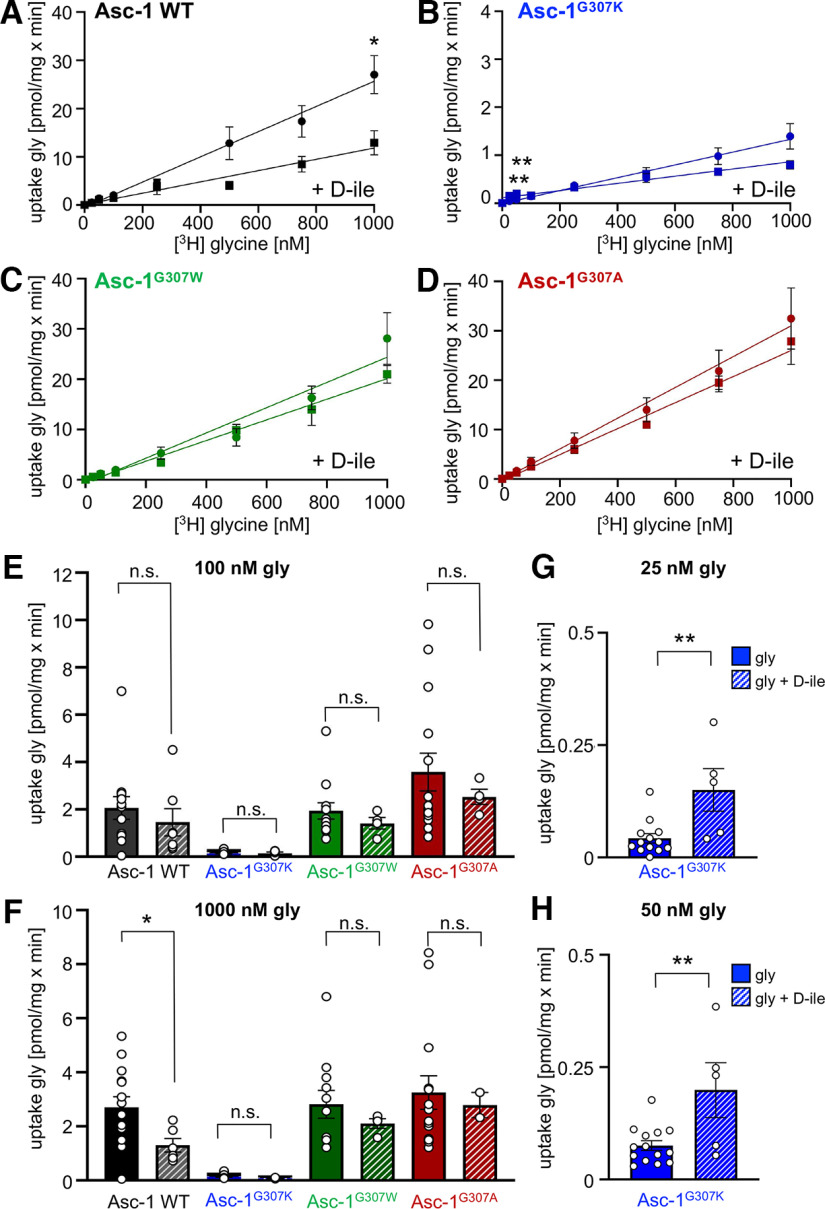
D-isoleucine is unable to inhibit Asc-1 glycine transport capacity in Asc-1 mutants at residue 307. ***A–D***, Glycine uptake in the presence and absence of 4 mm D-isoleucine (D-ile) for Asc-1 WT, and Asc-1 variants G307K, G307W, and G307A. In contrast to Asc-1 WT, the transport capacity was unchanged for all Asc-1 variants in the presence of D-ile. ***E***, Comparison of glycine uptake in the absence and presence of D-ile at low (100 nm) and (***F***) high glycine (1000 nm) concentration. ***G***, ***H***, Inhibition of glycine transport by D-ile (4 mm) at glycine concentrations 25 nm (***G***) and 50 nm (***H***) for Asc-1^G307K^. Four experiments have been performed (*n* = 4). Significance values: *p < 0.05, **p < 0.01.

Besides the novel identified Asc-1 mutation leading to a lack of Asc-1 function, we explored several artificial mutations at position 307. Asc-1^G307W^ allowed us to determine whether a general structural change from the small glycine toward a bulky, aromatic residue such as tryptophan might also disrupt Asc-1 function. In addition, we exchanged the glycine with a lysine (Asc-1^G307K^) which is highly similar and has the same positive charge within the side chain to the arginine observed in the patient. Moreover, we mutated glycine to alanine another amino acid with a small side chain (Asc-1^G307A^) and present in other LATs at the corresponding amino acid position. All Asc-1 variants showed expression in transfected HEK293 cells (Fig. 5*A*). Interestingly, the Asc-1^G307K^ lost transport activity at all glycine concentrations used similar to Asc-1^G307R^ (100 nm Asc-1 WT 2.066 ± 0.478 pmol/mg × min compared with Asc-1^G307K^ 0.140 ± 0.025 pmol/mg × min, *p* = 0.0081, one-way ANOVA). Surprisingly, both Asc-1^G307A^ and Asc-1^G307W^ with the bulky tryptophan preserved glycine transport capability and were indistinguishable from Asc-1 WT (Fig. 5*B–D*; Table 4). Inhibition of glycine transport was investigated for these mutants using D-isoleucine. All three Asc-1 variants Asc-1^G307K^, Asc-1^G307A^, and Asc-1^G307W^ were not blocked in their glycine transport activity at any glycine concentration used in the presence of D-isoleucine similar to the patient mutation Asc-1^G307R^ (Fig. 6*A–F*). Curiously, for Asc-1^G307K^ significantly increased transport activity was exhibited in the presence of D-isoleucine at glycine concentrations of 25–50 nm (Fig. 6*G*,*H*). By contrast, Asc-1 WT was again significantly blocked on presence of 4 mm D-isoleucine at 1000 nm glycine (*p* = 0.038) and decreased in transport capability of glycine at all lower glycine concentrations (25–750 nm (Fig. 6*A*,*F*; Table 4). Taken together, our data point to the importance of the amino acid residue glycine 307 localized in helix 10 of the Asc-1 transporter or the structural surrounding of glycine 307 for the binding of the inhibitor D-isoleucine and/or the process of functional Asc-1 transporter inhibition.

**Table 4.** Uptake capacity of Asc-1 variants

	[^3^H] glycine (nm)	Uptake					
		[^3^H] glycine (pmol/mg × min)		Significance	D-isoleucine (pmol/mg × min)		Significance
		Mean ± SEM	*n*	*p*-value compared with Asc1 WT	Mean ± SEM	*n*	*p*-value compared with (-) inhibitor
Asc1 WT	0	0.000 ± 0.000	14		0.000 ± 0.000	7	
	25	0.540 ± 0.084	14		0.440 ± 0.140	7	0.5253
	50	1.385 ± 0.337	14		1.087 ± 0.507	7	0.6220
	100	2.066 ± 0.478	13		1.461 ± 0.574	7	0.4467
	250	4.563 ± 0.831	13		3.676 ± 1.517	7	0.5813
	500	12.826 ± 3.365	13		4.037 ± 0.859	6	0.1006
	750	17.346 ± 3.255	12		8.465 ± 1.628	7	0.0640
	1000	27.047 ± 3.937	14		12.929 ± 2.507	6	0.0381
Asc1^G307K^	0	0.000 ± 0.000	14		0.000 ± 0.000	5	
	25	0.042 ± 0.010	13	<0.0001	0.150 ± 0.048	5	0.0047
	50	0.075 ± 0.011	14	0.0027	0.199 ± 0.061	5	0.0058
	100	0.140 ± 0.025	14	0.0081	0.157 ± 0.044	5	0.7321
	250	0.372 ± 0.073	14	0.0076	0.328 ± 0.048	5	0.7335
	500	0.526 ± 0.093	13	0.0003	0.608 ± 0.136	5	0.6431
	750	0.982 ± 0.174	13	<0.0001	0.657 ± 0.077	5	0.2770
	1000	1.395 ± 0.262	14	<0.0001	0.798 ± 0.089	4	0.2543
Asc1^G307W^	0	0.000 ± 0.000	14		0.000 ± 0.000	4	
	25	0.509 ± 0.092	14	0.8846	0.587 ± 0.051	4	0.6670
	50	1.224 ± 0.404	14	0.9053	0.946 ± 0.134	4	0.7248
	100	1.941 ± 0.347	13	0.9764	1.420 ± 0.249	4	0.4371
	250	5.295 ± 1.208	14	0.9968	3.464 ± 0.650	4	0.4438
	500	8.409 ± 1.735	13	0.3682	9.857 ± 1.159	4	0.6625
	750	16.288 ± 2.374	14	0.8666	13.980 ± 3.164	3	0.6758
	1000	28.107 ± 5.144	11	0.9992	20.983 ± 1.780	4	0.4324
Asc1^G307A^	0	0.000 ± 0.000	14		0.000 ± 0.000	4	
	25	0.667 ± 0.097	13	0.8138	0.688 ± 0.014	4	0.9078
	50	1.657 ± 0.324	14	0.9803	1.311 ± 0.056	4	0.5835
	100	3.590 ± 0.800	14	0.1323	2.535 ± 0.319	4	0.5029
	250	7.779 ± 1.582	14	0.1488	5.990 ± 0.806	4	0.5659
	500	14.019 ± 2.436	14	0.9814	11.006 ± 0.766	4	0.5283
	750	21.897 ± 4.251	12	0.8195	19.496 ± 1.377	4	0.7558
	1000	32.527 ± 6.209	14	0.9222	27.872 ± 4.681	2	0.7879

*n* = number of independent experiments.

### Structural importance of glycine 307

**Figure 7.**
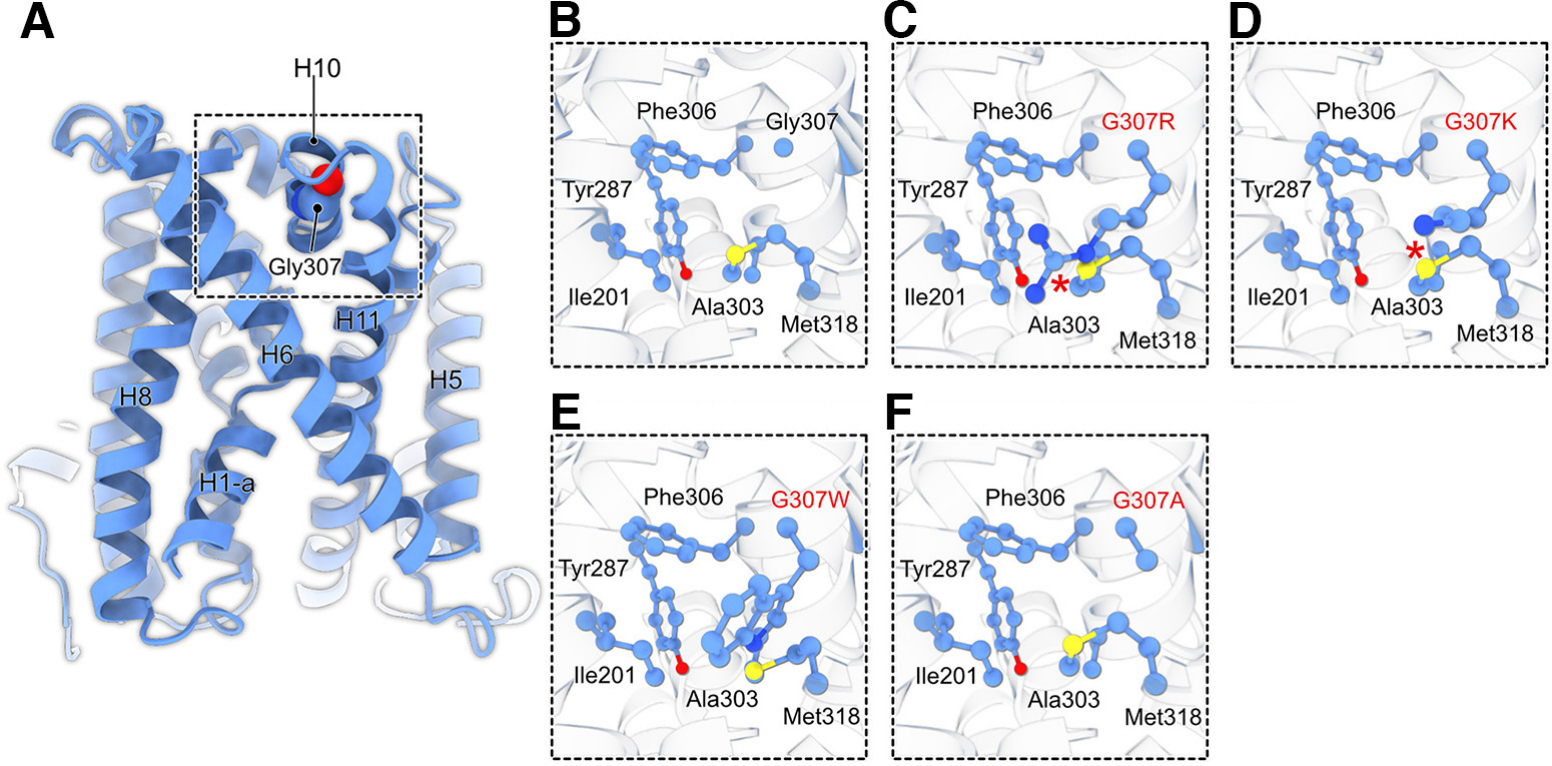
Modeling of the *SLC7A10* variants. ***A***, Overall architecture of the model predicted for human *SLC7A10* as predicted by AlphaFold2. In the model, sequences from 1 to 34 and 494 to 523 are not shown for easier representation. ***B–F***, Enlarged views of the environment surrounding of G307 in the unmodified WT sequence compared with G307R, G307K, G307W, and G307A.

Structural analysis of the predicted model and *in silico* mutational analysis showed that in Asc-1 WT, Gly307 in helix 10, resides at the extracellular end of the juxtamembrane region, surrounded by residues Ile201 (helix 6), Tyr287 (helix 8), Ala303 (helix 10), Phe306 (helix 10), and Met318 (helix 11), which form a hydrophobic core around Gly307 (Fig. 7*A*,*B*). Mutations of Gly307 to a charged bulkier residue either Arg (patient mutation) or to Lys create an uncomplimentary environment to occupy a hydrophobic pocket, which might explain the effects seen in the cellular assays. In addition, mutation into Arg or Lys might also clash with Met318, which might also impart a local structural rearrangement, which ultimately have impact on the transport activity of the transporter. By contrast, mutation of Gly307 to hydrophobic residues (Trp or Ala), in turn complement the nature of the bordering residues, thus might underline the negligible effect of these changes (Fig. 7*B–F*).

## Discussion

Startle disease is a rare neurologic disorder with disturbed glycinergic neurotransmission because of genetic variants in glycine receptors, transporters, or synaptically associated proteins of the receptor complex (Bode and Lynch, 2014; Schaefer et al., 2022). Within our patient cohort several patients diagnosed for startle disease do not carry a mutation in the known common genes, i.e., *GLRA1*, *GLRB*, *SLC6A5*. Here, we screened 51 patients from our startle disease cohort and identified *SLC7A10* (Asc-1) as a novel gene associated with human startle disease. From murine studies, *SLC7A10* (Asc-1) has been recently suggested as a possible additional candidate gene, since *Slc7a10* KO mice suffer from startle disease-like symptoms including increased tremor, hind-leg clasping and significantly enhanced righting time (Safory et al., 2015). Moreover, KO of Asc-1 was associated with a marked decrease in glycine levels in spinal cord and brainstem. The impairment in inhibitory neurotransmission was also shown at the functional level using brainstem slices characterized by significantly lower frequencies of spontaneous IPSCs but similar current amplitudes. Interestingly, the phenotype was rescued by replenishing glycine levels (Safory et al., 2015).

We discovered three exonic SNVs including one missense and two synonymous changes, seven intronic nucleotide variations, and 5′ UTR and 3′ UTR variations. Intronic mutations may affect splicing processes (Baralle and Baralle, 2005; Anna and Monika, 2018). An intronic mutation at a splice site has been shown to provoke aberrant splicing processes of the GlyR β subunit underlying startle disease in the *spastic* mouse, accompanied by lethality of homozygous animals at the age of three to four weeks (Mülhardt et al., 1994). In general, mutations in the 3′ UTR and 5′ UTRs have been demonstrated to affect mRNA levels and protein expression (Dvir et al., 2013; Lim et al., 2021). In *GLRB*, a genome-wide GWAS study identified SNVs in intronic and UTR regions which could be related to fear and panic disorders. Altered protein expression of GlyR β as a possible consequence of the SNVs has been suggested to account for the observed panic phenotype associated with enhanced startle responses in human (Deckert et al., 2017). Whether the identified intronic, 5′ and 3′ UTR variations in Asc-1 identified in the present study affect Asc-1 splicing or expression level needs further detailed investigation.

The two synonymous mutations represent common Asc-1 SNVs in the human population and therefore do most likely not underlie the pathology in startle disease (gnomAD, T358T, T486T). The identified missense mutation G307R in the patient although heterozygous is associated with a typical clinical picture of startle disease, prominent after birth. The patient suffered from muscle spasms and rigidity. During childhood at the age of two years, the motor phenotype was paralleled by a developmental delay suggesting that both inhibitory and excitatory pathways were affected at the molecular level in line with the dual role of Asc-1 in excitatory and inhibitory processes (Helboe et al., 2003; Rosenberg et al., 2013; Safory et al., 2015; Ehmsen et al., 2016; Sason et al., 2017). Unfortunately, a segregation analysis could not have been performed. Furthermore, we cannot exclude that other mutations in the genome may also lead to a startle disease-like phenotype.

We were however able to explain the observed clinical picture in the affected individual by detailed molecular, cellular, and functional investigation. Using an *in silico* structural prediction, we found that positively charged arginine or lysine residues at position 307 instead of a glycine form an uncomplimentary environment to occupy a hydrophobic pocket in transmembrane domain 10 of Asc-1. Positively charged residues at position 307 might also clash with methionine 318 leading to local structural rearrangements and thus altering the transport activity. In line with this structural prediction, the patient mutation G307R as well as an alternative lysine at position 307 within the Asc-1 transporter sequence led to a lack of transport capability and thus loss of function.

**Figure 8.**
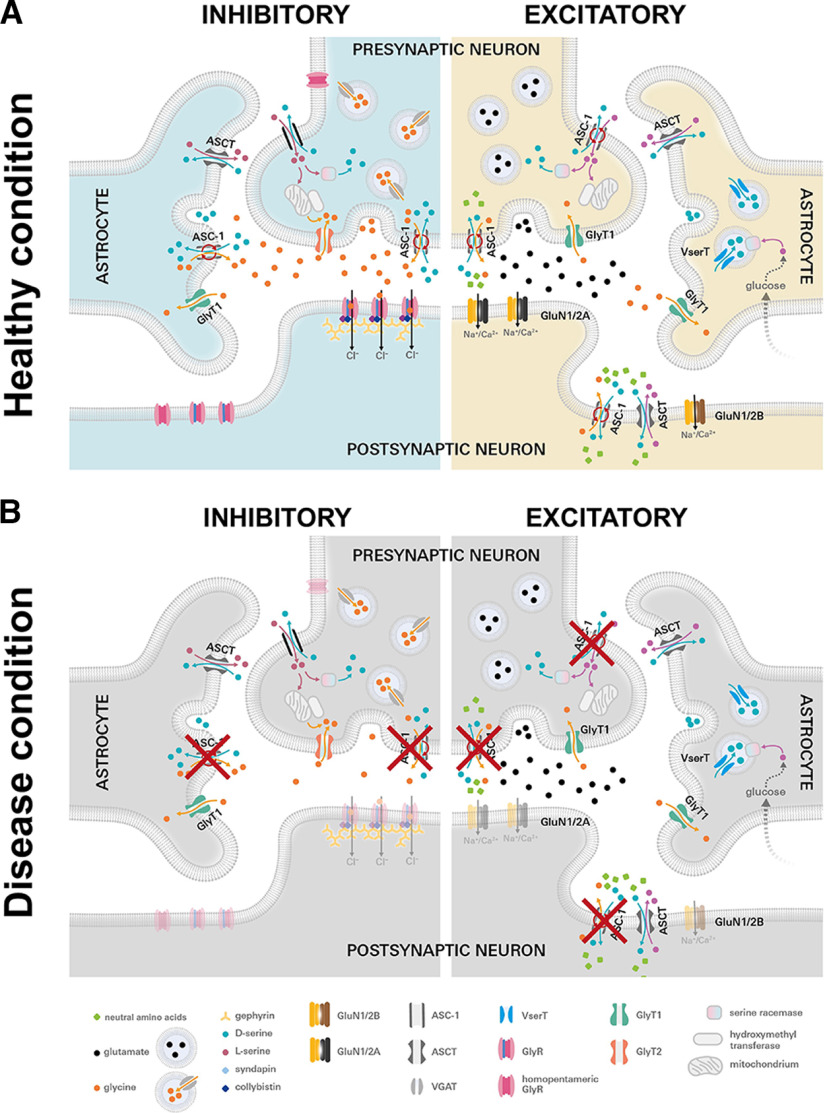
Glycine homeostasis at inhibitory and excitatory synapses and impact on disease patterns. ***A***, Glycinergic inhibitory and glutamatergic excitatory synapses are shown under healthy conditions. GlyRs at inhibitory synapses are either homomeric (presynaptic and extrasynaptic) and heteromeric (synaptic; pink) receptors clustered by gephyrin (yellow). The protein complex further interacts with collybistin (blue) and syndapin I (green). At presynapses, the glycine transporter GlyT2 (orange) transports back glycine (yellow circles) to be packed into vesicles for a second release. Asc-1 (gray) located at presynaptic membranes takes up L-serine (pink circles) released from astrocytes and transports out D-serine (blue circles). At the presynaptic terminal, Asc-1 is supposed to release glycine and takes up D-serine. Note, at neighboring astrocytes, Asc-1 determines glycine release and transports D-serine back into the astrocyte. The opposite directions of amino acid transport for Asc-1 are also described (marked by red arrows). At excitatory synapses, postsynaptic heteromeric NMDAR are localized [GluN1/2A (yellow/black) mainly at synaptic sites, at extrasynaptic sites mainly GluN1/2B (yellow/brown)]. Glutamate is the main neurotransmitter (black circles). Instead of GlyT2, at excitatory synapses GlyT1 (green) is localized presynaptically and in astrocytic membranes. Asc-1 is also located at presynaptic and postsynaptic membranes, transporting D-serine (blue circles), glycine (yellow circles), and neutral amino acids (green rectangles). ***B***, Inhibitory and excitatory synapse under disease condition. If Asc-1 is functionally impaired (red crosses), less synaptic glycine is available resulting in malfunction of postsynaptic GlyRs (shown transparent) and thus in startle disease (left image). At excitatory synapses, nonfunctional Asc-1 (red crosses) leads to less availability of D-serine/glycine which are both essential co-agonists at the NMDAR complex thereby reducing excitatory synaptic signal propagation (demonstrated by transparent NMDAR subtypes (GluN1/2A and GluN1/2B). Impaired excitatory neurotransmission is compatible with developmental delay in patients.

In contrast to functional impairment, the missense mutation G307R in the Asc-1 transporter did not result in altered expression levels of Asc-1. Therefore, the underlying mechanism of glycinergic dysfunction is different from significant reductions in GlyR levels often seen in hyperekplexia patients carrying recessive mutations in the GlyR α1 or β subunits (Bode et al., 2013; Schaefer et al., 2015). Asc-1^G307R^ expression level were only slightly reduced compared with Asc-1 WT in whole-cell lysates and subfractions of surface membranes. Thus, the mutation G307R did not interfere with trafficking of the mutated Asc-1 together with its heavy chain 4F2hc to the cell surface. It has been previously shown that the heavy chain 4F2hc covers the extracellular surface of the light chain and thereby stabilizes Acs-1 at the membrane (Rosell et al., 2014). Rather, functional investigation of the mutated Asc-1 transporter showed a loss of glycine uptake comparable to that seen in *Slc7a10* KO mice (Rutter et al., 2007; Safory et al., 2015). Hence, the observed motor symptoms in our patient can be explained by the expressed nonfunctional Asc-1 at inhibitory synapses (Fig. 8*A*). Nonfunction has also been observed for dominant mutations in GlyR α1 (Bode and Lynch, 2014). Most GlyT2 mutations that result in loss of function are homozygous, or compound heterozygous (Harvey et al., 2008). Nonfunction of Asc-1 was also exhibited for G307K but not for G307A or G307W which showed transport activity indistinguishable from WT Asc-1. Interestingly, the introduction of a bulky tryptophan harboring an aromatic ring structure complement the nature of the bordering residues suggesting a silent effect accompanied with this exchange. The unaltered function of G307A can be explained by the restoration of the hydrophobic core by the alanine. Interestingly, the homologous amino acid position to G307 among human LATs is mainly occupied by an alanine, e.g., in the bacterial alanine-serine-cysteine exchanger (BasC), the human aspartate/glutamate transporter 1 (hAGT1), and the bacterial glutamate/cystine antiporter (ApcT) and L-arginine/agmatine transporter (AdiC; Errasti-Murugarren et al., 2019), supporting the normal function for Asc-1^G307A^. We conclude that the hydrophobic pocket formed around residue 307 is an essential component of Asc-1 function.

Asc-1 function can be blocked by D-isoleucine, which mediates Asc-1 glycine uptake but does not affect glycine transporters GlyT1/GlyT2, or neuronal/astrocytic glutamate uptake (Rosenberg et al., 2013). We found that residue G307 of Asc-1 is an important position mediating the blocking effect of D-isoleucine at Asc-1. None of our mutants displayed D-isoleucine inhibition of glycine uptake at low or high glycine concentrations. Rather, an increased glycine transport was obtained at low glycine concentrations (25–50 nm) for the mutant G307K. Therefore, we propose that G307 is a key determinant for conformational transitions of Asc-1 that allow amino acid transport across a membrane. Conformational transitions between an outward-open conformation, an outward-open-occluded conformation, and an inward-open conformation include uptake and efflux modes of Asc-1 which differ in affinity and velocity (Mikou et al., 2020).

Amino acid uptake and efflux by Asc-1 is important for glycine homeostasis in the central nervous system and essential for inhibitory and excitatory neurotransmission processes. Glycine represents an agonist at inhibitory glycine receptors finally coordinating muscle activity important for motor behavior and respiratory processes (Mesuret et al., 2018; Schaefer et al., 2022) and an essential coagonist at excitatory NMDA receptors enabling processes such as memory, learning, and synaptic plasticity (Martineau et al., 2014). Asc-1 is expressed in a subset of astrocytes of the spinal cord (Ehmsen et al., 2016) and enables glycine transport opposite to D-serine transport, providing a substrate for GlyT2 which takes glycine up into glycinergic presynapses. L-serine uptake by Asc-1 at presynaptic membranes enables further glycine synthesis via the serine-hydroxymethyl transferase (Fig. 8*A*). Dysfunction of Asc-1 displays low glycine levels with consequently decreased inhibitory neurotransmission such as that observed in startle disease.

At excitatory synapses (Fig. 8*B*), lack of Asc-1 suggests less availability of glycine as essential co-agonist for the activation of excitatory NMDA receptors. Glycine as coagonist is more important at extrasynaptic sites activating GluN1/2B receptors while at synaptic sites the GluN1/2A receptor composition is the major subunit combination using preferentially D-serine (Papouin et al., 2012). D-serine is mainly provided by neurons dependent on astrocytic export of L-serine and intracellular glycine level however astrocytic release of D-serine cannot be excluded (Rosenberg et al., 2010; Martineau et al., 2014; Neame et al., 2019). In contrast to GlyT1 which enables efficient glycine uptake, Asc-1 in this context is important for glycine/D-serine transport. Hence, the developmental delay observed in our proband might be explained by lack of Asc-1 transport activity not providing enough glycine/D-serine for proper function of excitatory synapses in the critical period of synaptic network and plasticity formation. Hence, our data further support the recently suggested synergistic control of glycine turnover by Asc-1 together with GlyT2 at inhibitory synapses and with GlyT1 at excitatory synapses (Eulenburg and Hulsmann, 2022).

In conclusion, this is the first description of a pathogenic variant in *SLC7A10* encoding Asc-1, highlighting the importance of this gene in human startle disease associated with developmental delay. Depending on the severity of the Asc-1 impairment, inhibitory or excitatory pathways might be preferentially affected or alterations in both signal transduction processes may underlie this distinct disease pattern.

## Synthesis

Reviewing Editor: Michael Michaelides, NIDA-NIH Decisions are customarily a result of the Reviewing Editor and the peer reviewers coming together and discussing their recommendations until a consensus is reached. When revisions are invited, a fact-based synthesis statement explaining their decision and outlining what is needed to prepare a revision will be listed below. The following reviewer(s) agreed to reveal their identity: NONE. Note: If this manuscript was transferred from JNeurosci and a decision was made to accept the manuscript without peer review, a brief statement to this effect will instead be what is listed below.

Reviewer 1

The changes are adequate. The specific nature of startle disease and the clear symptoms in the index case are a compelling evidence for the role of Slc7a10 in disease

Reviewer 2

This is a modified version of a previous paper in which I raised some concerns, which have been adequately answered by the authors.
